# A new ferroptosis-related genetic mutation risk model predicts the prognosis of skin cutaneous melanoma

**DOI:** 10.3389/fgene.2022.988909

**Published:** 2023-01-05

**Authors:** Jia He, Wenting Huang, Xinxin Li, Jingru Wang, Yaxing Nie, Guiqiang Li, Xiaoxiang Wang, Huili Cao, Xiaodong Chen, Xusheng Wang

**Affiliations:** ^1^ School of Pharmaceutical Sciences (Shenzhen), Sun Yat-Sen University, Guangzhou, China; ^2^ Department of Burn Surgery, The First People’s Hospital of Foshan, Foshan, China; ^3^ CAS Key Laboratory of Molecular Virology and Immunology, Institut Pasteur of Shanghai, Chinese Academy of Sciences, Shanghai, China

**Keywords:** skin cutaneous melanoma, ferroptosis, genetic mutation, single nucleotide variant, prognosis, tumor immunity

## Abstract

**Background:** Ferroptosis is an iron-dependent cell death mode and closely linked to various cancers, including skin cutaneous melanoma (SKCM). Although attempts have been made to construct ferroptosis-related gene (FRG) signatures for predicting the prognosis of SKCM, the prognostic impact of ferroptosis-related genetic mutations in SKCM remains lacking. This study aims to develop a prediction model to explain the relationship between ferroptosis-related genetic mutations and clinical outcomes of SKCM patients and to explore the potential value of ferroptosis in SKCM treatment.

**Methods:** FRGs which significantly correlated with the prognosis of SKCM were firstly screened based on their single-nucleotide variant (SNV) status by univariate Cox regression analysis. Subsequently, the least absolute shrinkage and selection operator (LASSO) and Cox regressions were performed to construct a new ferroptosis-related genetic mutation risk (FerrGR) model for predicting the prognosis of SKCM. We then illustrate the survival and receiver operating characteristic (ROC) curves to evaluate the predictive power of the FerrGR model. Moreover, independent prognostic factors, genomic and clinical characteristics, immunotherapy, immune infiltration, and sensitive drugs were compared between high—and low—FerrGR groups.

**Results:** The FerrGR model was developed with a good performance on survival and ROC analysis. It was a robust independent prognostic indicator and followed a nomogram constructed to predict prognostic outcomes for SKCM patients. Besides, FerrGR combined with tumor mutational burden (TMB) or MSI (microsatellite instability) was considered as a combined biomarker for immunotherapy response. The high FerrGR group patients were associated with an inhibitory immune microenvironment. Furthermore, potential drugs target to high FerrGR samples were predicted.

**Conclusion:** The FerrGR model is valuable to predict prognosis and immunotherapy in SKCM patients. It offers a novel therapeutic option for SKCM.

## Introduction

Skin cutaneous melanoma (SKCM), which is the most aggressive skin cancer, takes up for more than 75% mortality rate of skin-related cancers. Although patients with localized and regional cutaneous melanoma have a 5-year relative survival of 98% and 64% respectively. Once metastasized through the body, the 5-years survival rate falls to 23% (Rebecca et al., 2020). Generally, surgical resection is considered the first choice for patients with early-stage disease. Moreover, some advanced melanoma is insensitive to radiotherapy and chemotherapy as for its high aggressiveness (Ping et al., 2022). Therefore, several therapeutic agents including kinase inhibitors and immune checkpoint inhibitors (ICIs) were developed (Leonardi et al., 2018; Leonardi et al., 2020). Nowadays, Immunotherapy and targeted therapy have shown promising results in clinical trials and become the backbone of systemic treatment (Pelster and Amaria, 2019; Ribas et al., 2019). Despite the rapid development of these therapeutic approaches, limitations emerged since SKCM is heterogeneous cancer. Patients with the same stage and treatments may have a different prognosis and treatment response (Ackerman et al., 2014; Hassel et al., 2016; Simeone et al., 2017). Therefore, it is crucial to identify a prognostic predictive biomarker to inform clinical prognosis and treatment response.

Ferroptosis which was discovered in recent years is a novel form of programmed cell death and is characterized by a large amount of iron accumulation and lipid peroxidation (Li et al., 2020). It differs from other forms of cell death such as apoptosis, pyroptosis, necroptosis, and autophagy in morphology, biochemistry, and genetics (Gao et al., 2016). The main mechanism of ferroptosis is phospholipid peroxidation, which relies on the transition metal iron, reactive oxygen species (ROS), and phospholipids. In addition, nutrients, intra/intercellular signaling, and environmental stresses contribute to ferroptosis by regulating cellular metabolism and ROS levels (Jiang et al., 2021). Increasing evidence has indicated that ferroptosis was closely associated with the tumorigenesis and progression of cancers (Li et al., 2020). Many tumor suppressors show susceptibility to ferroptosis. Hence, regulating the antitumor activity of these tumor suppressors could be explored as an anticancer therapy (Jiang et al., 2021). Furthermore, Erastin, Sulfasalazine, Sorafenib, and other small molecule ferroptosis inducers used in the clinical treatment of cancer showed promising outcomes of anti-tumor effect (Liang et al., 2019; Xu G et al., 2021). Recent studies investigated that the differentiation status of melanoma cells was correlated with the susceptibility to ferroptosis. Ferroptosis inducers could decrease the number of dedifferentiated melanoma cells and prevent their immunosuppressive actions (Rebecca et al., 2020; Ping et al., 2022) (Gagliardi et al., 2020; Talty and Bosenberg, 2022). Apart from ferroptosis inducers, some miRNAs and genes associated with ferroptosis are involved in the development of SKCM. A previous study reported that miR-137 acts as a negative regulator of ferroptosis by directly targeting glutamine transporter SLC1A5 in melanoma cells (Luo et al., 2018). Additionally, miR-9 suppressed Erastin- and RSL3-induced ferroptosis by targeting glutamic-oxaloacetic transaminase GOT1 in melanoma cells (Zhang et al., 2018). Inhibiting mitochondrial complex I induced autophagosome formation, mitophagy, a cytosolic ROS increase and ultimately lead to necroptosis/ferroptosis in melanoma cells (Basit et al., 2017). Besides, evidence suggested that GPX4, VDAC2/3, NEDD4, AKRs, and SLC7A11 are involved in the resistance to ferroptosis in melanoma (Talty and Bosenberg, 2022). Ferroptosis has been a new hope for SKCM therapeutics. Nevertheless, the roles of ferroptosis-related genes in prognostic prediction and tumor microenvironment (TME) remain unclear.

Recent studies have consistently revealed biomarkers such as tumor mutation burden (TMB), neoantigen load (NAL), programmed cell-death receptor 1 ligand (PD-L1) expression, and lactate dehydrogenase (LDH) to predict therapeutic benefit in SKCM (Jiang J et al., 2020). Unfortunately, there still existed several limitations to their clinical application, including the undefined cut-off value, intra/intratumor heterogeneity, unsatisfactory predictive power, and relatively high cost (Jiang J et al., 2020; Bai et al., 2020). This highlights more effective and clinically actionable biomarkers are required to be identified.

Genetic mutations are heritable changes in the nucleotide sequence of DNA that resulted from both inherited and environmental factors. The mutator phenotype hypothesis suggests that the capacity to divide, invade, and metastasize of cancer cells results from genetic mutations that maintain the stability of genes in normal cells. Mutations in genetic stability genes initiate mutations by causing mutations in other genes that govern genetic stability. Next, some of the resulting mutated cells expand and achieve clonal dominance (Loeb et al., 2003). Notably, targeted therapy based on the specific genetic background has made a great progress. For example, BRAF mutations were discovered in nearly half of metastatic SKCM. Patients with BRAF mutations showed improved progression-free survival by treatment with two BRAF inhibitors vemurafenib and dabrafenib (Hauschild et al., 2012; Jin et al., 2019). However, the mutations in cancers affect drug sensitivity and drive drug resistance. Therefore, the outcomes of targeted therapy are largely dependent upon the mutation profile of tumors in patients.

In this study, we performed comprehensive analysis utilizing data downloaded from TCGA and GEO databases, along with FRGs identified in previous studies to determine potential ferroptosis-related prognostic genes of SKCM in accordance with SNV mutational status. Subsequently, we developed and evaluated a ferroptosis-related genetic mutation risk (FerrGR) model for predicting prognosis and assessing multiple roles of ferroptosis-related genetic mutations in the TME of SKCM. In addition, an integrated prognostic nomogram was established by combining the risk model and clinicopathological features to ameliorate the prognostic assessment of SKCM patients. We also characterized the distinctive immune landscape and genetic and epigenetic signature associated with the FerrGR model. Besides, potential drugs were predicted in the light of the FerrGR score. Overall, the FerrGR model might provide an effective prediction tool and help guide clinical decisions on therapy for SKCM.

## Materials and methods

### Data collection

All datasets used in this study were publicly available. RNA-seq transcriptome data, somatic mutations, SNVs, copy number variations (CNVs), methylation, clinical characteristics, and survival information were downloaded from The Cancer Genome Atlas (TCGA) database (http://www.cgga.org.cn/) and the Gene-Expression Omnibus (GEO) database (GSE91061). TMB data of Pan-Cancer was received from the GDC database. 63 immune checkpoint marker genes were obtained from the literature (Hu et al., 2020). A total of 299 ferroptosis-related genes were obtained from the FerrDb database (http://www.zhounan.org/ferrdb/) and a literature search (Liang et al., 2020; Zhuo et al., 2020; Hong et al., 2021; Tang et al., 2021). Among them, the TCGA-SKCM cohort contains 286 ferroptosis-related genes which were selected for further analysis (Supplementary Table S1).

### Identification of the prognostic FRGs

SNV mutations of FRGs in the TCGA-SKCM cohort were counted by the “maftools” R package. The heatmap of FRGs was drawn by the “ComplexHeatmap” R package. Tumor patients in TCGA-SKCM cohorts were classified as the mutation and the wild-type based on the presence or absence of SNV mutations in FRGs. Thereafter, the prognostic value of FRGs was determined by univariate Cox regression analysis using the R package “survival” where *p* < 0.1 was considered statistically significant. “Forestplot” R package was used to plot the forest map of prognostic FRGs. “ggpubr” R package was used to plot the sample proportion pie chart of prognostic ferroptosis related-genes mutation/wild-type samples. “Survminer” and “Survival” R packages were used to plot the survival curve of mutation/wild-type patients.

### Establishment of a ferroptosis-related genetic mutation risk (FerrGR) model

TCGA-SKCM mutational cohorts were divided into training and validation cohorts with the ratio of the training: validation = 7:3. The prognostic risk characteristics were assessed using the “glmnet” and “survival” R package based on the LASSO method in the training cohort. The FerrGR score was calculated according to the SNV mutational status (SNV mutation was equivalent to 1, while wild-type status was 0) of the key FRGs and the corresponding regression coefficient. The computational formula was as follows:

$$FerrGR\;score=\sum LASSO\;regression\;coefficient\times SNV\;mutational\;value\;of\;key\;gene\;\left(0\;or\;1\right)$$

The “forestplotdrug-sensitive” R package was used to draw the forest map of the key genes included in the model and their coefficients in the model.

### Validation of the FerrGR model

The patients in the training cohort were divided into High—and Low—FerrGR groups according to the optimal threshold obtained by the “surminer” R package. Then, the SNV type and frequency of key genes in the training cohort were counted with the package “maftools”. In addition, the heatmap of FRGs was drawn by the package “ComplexHeatmap”, while the survival curves of the two subgroups were created by the package “survminer” and “survival”. Subsequently, the package “pROC” was used to calculate and draw the ROC curve of FerrGR, TMB, and MSI in the validation cohort.

### Construction of the predictive nomogram based on the FerrGR model

The clinical characteristics including sample type, tumor stage, gender, the value of Clark’s level, BMI, TNM-staging, and TCGA molecular typing in different subgroups of the FerrGR model were calculated by the R package “ggpubr”. The FerrGR scores and the clinical characteristics were inputted into univariate and multivariate Cox analysis to validate whether the FerrGR score was an independent risk factor for SKCM. After that, a nomogram was constructed by “regplot” and “rms” packages for predicting the progression of SKCM patients.

### Multiomics characteristics analysis

The different landscape of SNVs, amplification and deletion of FRGs between high—and low—FerrGR groups was identified by the chi-square test. In addition, the differential expression and genomic methylation of FRGs between subgroups were analyzed with the “limma” package.

### FerrGR model for immunotherapy

Data of SNV mutations from the dataset GSE90161 was scored by the FerrGR model, and then the patients were divided into high - and low - FerrGR groups based on the median value. After grouped, the heatmap of immune checkpoint genes and the survival curve of different groups were performed by “complexheatmap”, “survminer” and “survival” R packages, respectively. Besides, immunotherapeutic response PD (progressive disease)/SD (stable disease) and CR (complete response)/PR (partial response) was assessed by “ggstatsplot” package.

### Survival analysis on basis of the FerrGR model combined with TMB or MSI

We got the MSI status of patients from the TCGA-SKCM dataset by the “PreMSIm” package. Subsequently, patients were grouped into high—and low—MSI groups based on the median value. In combination with the FerrGR model, the patients were split into three groups: the first group’s scores in MSI and FerrGR model were both high, the second group’s scores were both low, and the third group’s scores were single high. The prognostic survival curve of these three groups was then analyzed and plotted by package “survminer” and “survival”. The prognostic survival analysis by the FerrGR model combined with TMB was done in the same way.

### Tumor microenvironment analysis

Here, we used package “estimate” to calculate stromal and immune scores for predicting the level of infiltrating stromal and immune cells, and the tumor purity was also inferred in TCGA-SKCM cohort patients. The differences in the clinical characteristics, FerrGR score, stromal and immune scores, and tumor purity between high—and low—FerrGR groups were then statistically analyzed, where the *t*-test and chi-square test were used for continuous and categorical variables respectively. Subsequently, the infiltration of immune cells in the TCGA-SKCM cohort was estimated using the cibersort algorithm. The R package “ggpubr” was performed to count the differential expression of immune checkpoints between the two subgroups. The gene set variation analysis (GSVA) was conducted to calculate the scores of enrichments in immune pathways by complying with the “GSEABase” and “GSVA” R package.

### Potential sensitive drug prediction

The drug sensitive information and corresponding expression were downloaded from the PRISM Repurposing 19Q4 dataset (https://depmap.org/portal/download/all/) and Cancer Therapeutics Response Portal v2.1 (https://ocg.cancer.gov/programs/ctd2/data-portal/#). In addition, SNVs and samples’ information of CCLE cell lines was obtained (https://depmap.org/portal/download/all/). Next, SKCM cell lines were divided into high—and low—FerrGR groups by calculated FerrGR score. Drug sensitivity of cell lines was qualified as an AUC value, and a lower AUC value suggested higher drug sensitivity. We then used the package “corrr” for exploring the correlations between the FerrGR score and AUC/IC50.

### Statistical analysis

The R software (version: 4. 0. 2) was utilized to conduct all the statistical analyses in this article. All *p* values of statistical data were based on two-sided statistical tests, and data with *p* < 0.05 was considered to be statistically significant (except for the univariate Cox proportional hazards regression model, where *p* < 0.1 was considered to be statistically significant).

## Results

### Identification of prognosis-related FRGs in the TCGA-SKCM cohort

**FIGURE 1:**
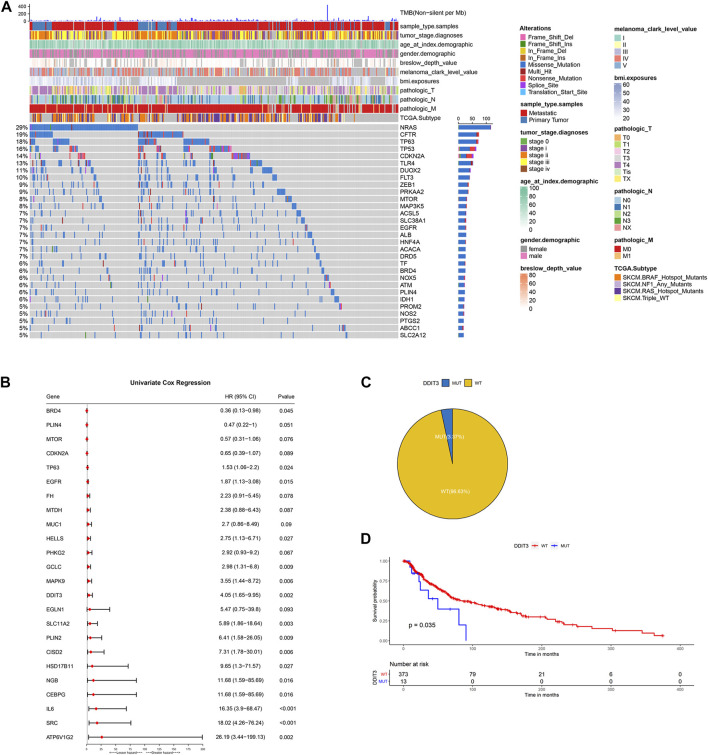
Identification of prognosis-related key FRGs in SKCM. **(A)** Heatmap to show the SNV landscape of the top 30 FGRs with the most frequent SNV mutations in the TCGA-SKCM cohort. **(B)** Forest plots showing the results of the univariate Cox regression analysis between FGRs and prognosis. **(C)** Pie charts depicting the proportions of wild-type and DDIT3-mutant patients. **(D)** Kaplan-Meier survival analysis of the wild-type and DDIT3-mutant patients.

The flowchart of the present research is shown in Supplementary Figure S1. A total of 463 SKCM patients from the TCGA-SKCM cohort were included in this study. The detailed clinical characteristics of these patients were summarized (Supplementary Table S2). We firstly identified the SNV landscape of 286 FRGs in SKCM patients. SNVs were discovered in most FRGs and the 30 top-ranked FRGs were present in the heatmap of Figure 1A. Of note, the top 2 highest ranked FGRs were NRAS and CFTR, which had 29 and 19 percent SNV mutation regions respectively. Subsequently, 24 prognosis-related genes were screened from all 286 FRGs by the univariate Cox regression analysis of overall survival (OS) (*p* < 0.1), shown in the forest plot (Figure 1B). According to the value of hazard ratio (HR), ATP6V1G2, SRC, IL6, CEBPG, and NGB were considered the genes with the highest risk. To examine the prognostic significance of these screened risk FRGs, DNA Damage Inducible Transcript 3 (DDIT3), one of the risk FRGs, was performed as an example. 3.37% of SKCM patients were observed to carry DDIT3 mutations and these patients significantly had worse OS than patients without DDIT3 mutations by Kaplan-Meier survival analysis (Figures 1C,D).

### Construction and validation of the ferroptosis-related genetic mutation risk (FerrGR) model

**FIGURE 2:**
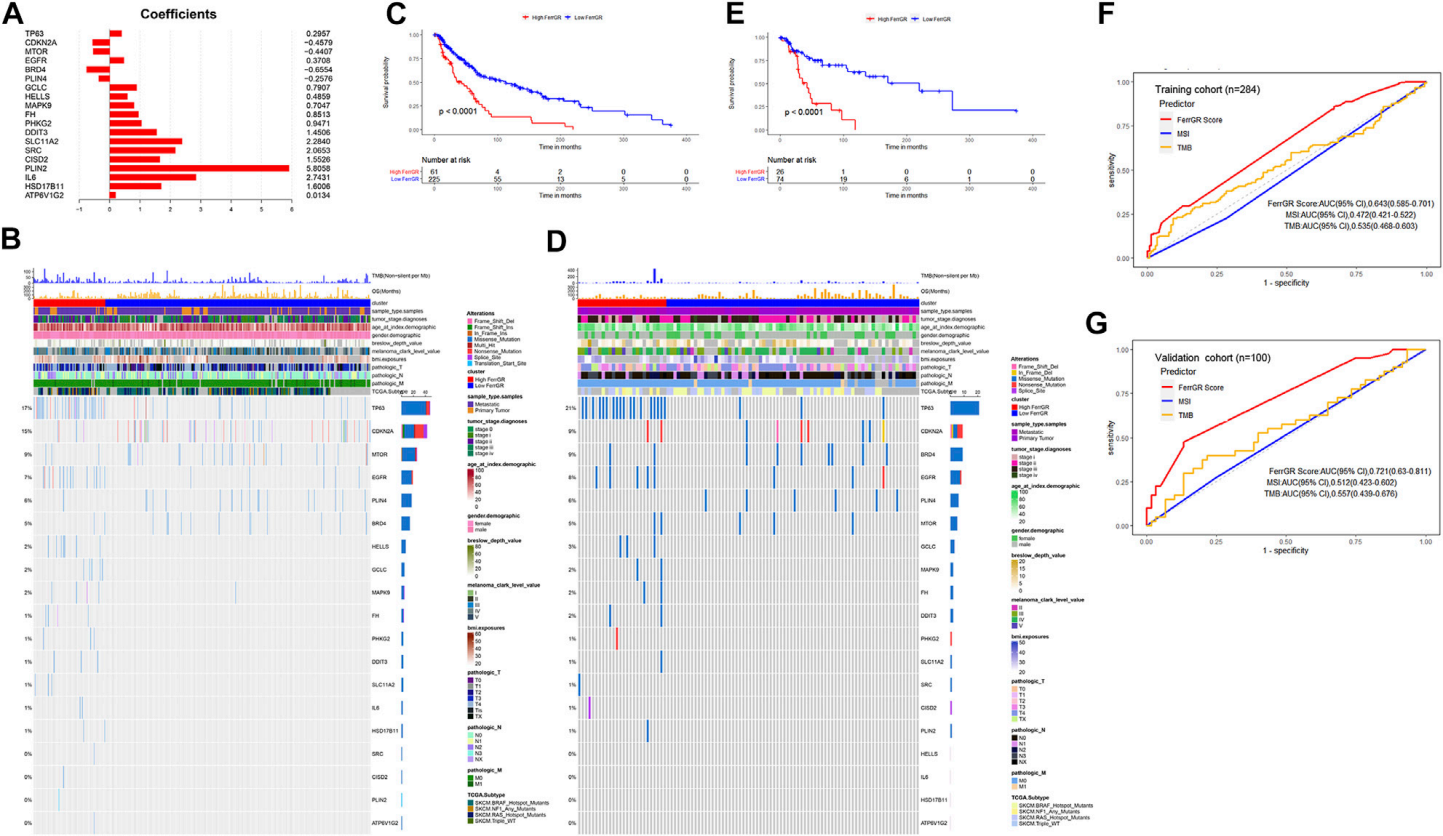
Construction and prognostic analysis of the ferroptosis-related genetic mutation risk (FerrGR)model in the training cohort and validation cohort. **(A)** Lasso coefficient spectrum of 19 key FRGs in the FerrGR model **(B,D)** SNV heatmap and clinicopathologic features of 19 key FRGs in the **(B)** training cohort and **(D)** validation cohort. **(C,E)** Kaplan-Meier curves of the FerrGR model for SKCM patients with different risk groups in the **(C)** training cohort and **(D)** validation cohort. **(F,G)** ROC analysis of the FerrGR model compared with TMB and MSI.

To prevent the risk of over-fitting, the LASSO Cox regression analysis was performed to establish a prognostic prediction model based on whether patients carrying SNV mutations in the above screened 24 FRGs or not. As a result, 19 key genes (TP63, CDKN2A, MTOR, EGFR, BRD4, PLIN4, GCLC, HELLS, MAPK9, FH, PHKG2, DDIT3, SLC11A2, SRC, CISD2, PLIN2, IL6, HSD17B11, ATP6V1G2) were filtered out by the minimum value of lambda (λ) (Supplementary Figure S2). The coefficients of these genes were shown in Figure 2A. The risk score was calculated with the following formula: 0.295704560557983 × SNV mutational value of TP63 + (−0.457905201754129) × SNV mutational value of CDKN2A + (−0.440669922200648) × SNV mutational value of MTOR + 0.370753348528944 × SNV mutational value of EGFR + (−0.655423174928644) × SNV mutational value of BRD4 + (−0.257562121098952) × SNV mutational value of PLIN4 + 0.790692660435278 × SNV mutational value of GCLC + 0.485910362202744 × SNV mutational value of HELLS + 0.70467787865497 × SNV mutational value of MAPK9 + 0.851337406732927 × SNV mutational value of FH + 0.947077530588054 × SNV mutational value of PHKG2 + 1.45060826783863 × SNV mutational value of DDIT3 + 2.28402264491982 × SNV mutational value of SLC11A2 + 2.06527868435253 × SNV mutational value of SRC + 1.55256390491241 × SNV mutational value of CISD2 + 5.80583718370437 × SNV mutational value of PLIN2 + 2.7431230119215 × SNV mutational value of IL6 + 1.60058988692493 × SNV mutational value of HSD17B11 + 0.0133531074086934 × SNV mutational value of ATP6V1G2.

The patients in the training cohort were classified into the high ferroptosis-related genetic mutation risk (high FerrGR) group and low ferroptosis-related genetic mutation risk (low FerrGR) group by the median risk score as a cut-off value, which was calculated as 0.2467727. The SNV landscape of 19 key FRGs in the TCGA training cohort was further figured out based on the two subgroups (Figure 2B). The Kaplan-Meier analysis indicated that patients in the high FerrGR group had significantly worse OS than those in the low FerrGR group (Figure 2C).

To test the reliability of the FerrGR model, the same formula as the training cohort was performed to calculate risk scores for the patients in the validation cohort. The patients were then allocated into the high FerrGR group and low FerrGR group by the same cut-off value. The SNV landscape of 19 key FRGs for patients in the validation cohort was shown in Figure 2D. Similar to the training cohort, The high FerrGR group exhibited a poorer survival outcome when compared to the low FerrGR group (Figure 2E).

Subsequently, we used the ROC curve to evaluate the prediction efficacy of the model by calculating the areas under the curve (AUC). The AUCs of the FerrGR model for one-year survival time were 0.643 in the training cohort and 0.721 in the validation cohort respectively; Besides, the FerrGR model showed the best prognostic power compared with TMB and MSI (Figures 2F,G).

### Correlations between the FerrGR score and clinicopathological factors

**FIGURE 3:**
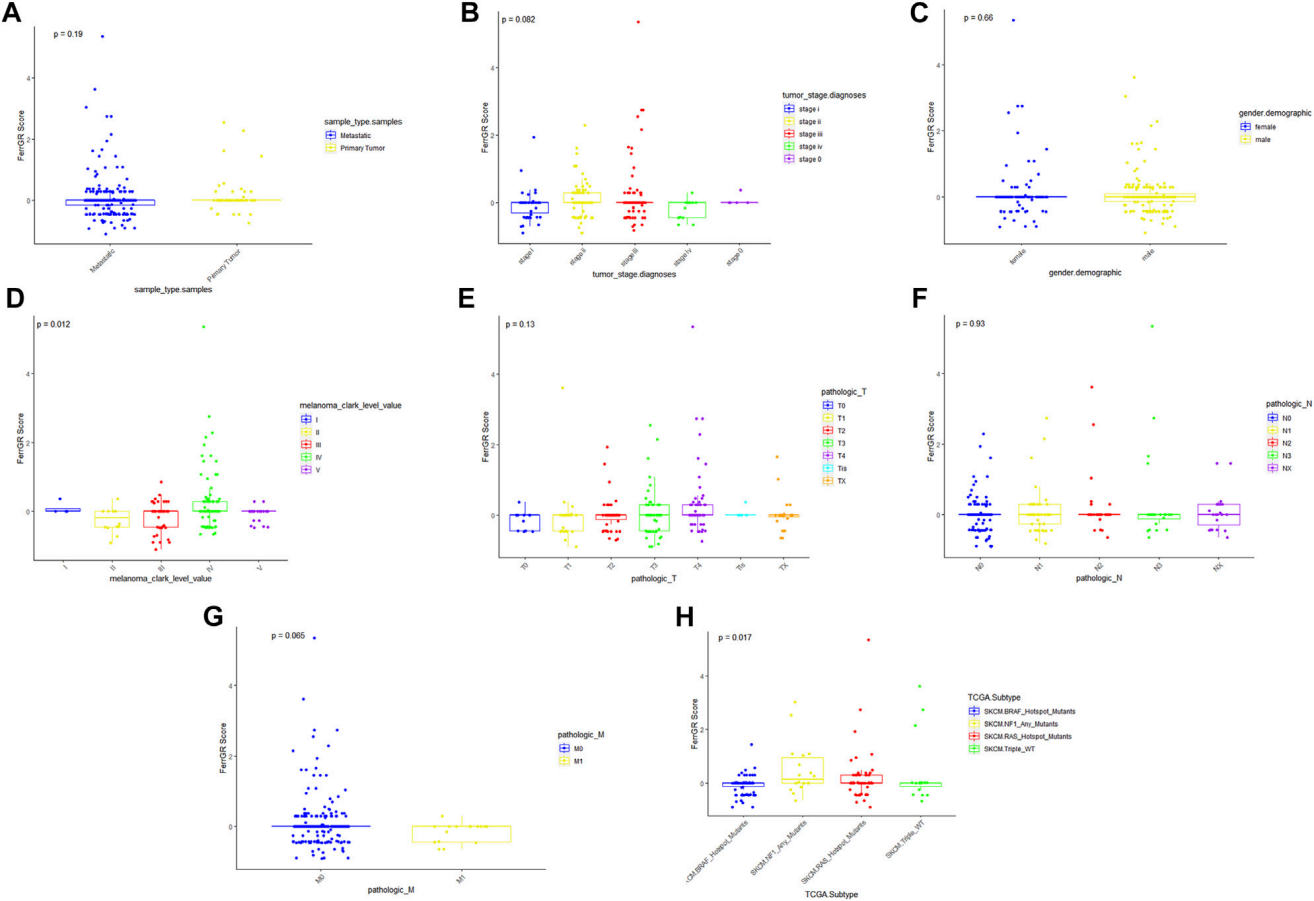
Relationships between the FerrGR score and clinicopathological features. The boxplots showed whether the FerrGR score was correlated with pathological features in SKCM patients, including **(A)** sample type, **(B)** tumor stage, **(C)** gender, **(D)** Clark level, **(E)** T stage, **(F)** N stage, **(G)** M stage, and **(H)** TCGA subtype.

To further explore the roles of the FerrGR model in the SKCM development, the correlations between the FerrGR score and clinicopathological factors were studied. Our results showed that the FerrGR score was independent with sample type (*p* = 0.19, Figure 3A), gender (*p* = 0.66, Figure 3C), T stage (*p* = 0.13, Figure 3E) and N stage (*p* = 0.93, Figure 3F). Further, there may be some correlation between the FerrGR score and tumor stage (*p* = 0.082, Figure 3B). The FerrGR score in stage II patients was higher than in other stages. Furthermore, The FerrGR score in stage M0 patients was higher than in the M1 stage (*p* = 0.065, Figure 3G). In particular, the FerrGR score was significantly among the values of Clark levels (*p* = 0.012, Figure 3D), and the signature was associated with TCGA subtypes (*p* = .017, Figure 3H). The FerrGR score in Clark level III patients was the highest. Besides, patients with NF1 mutations have a higher score than patients in other TCGA subtypes.

### Independent prognostic factors analysis and nomogram prediction model construction

**FIGURE 4:**
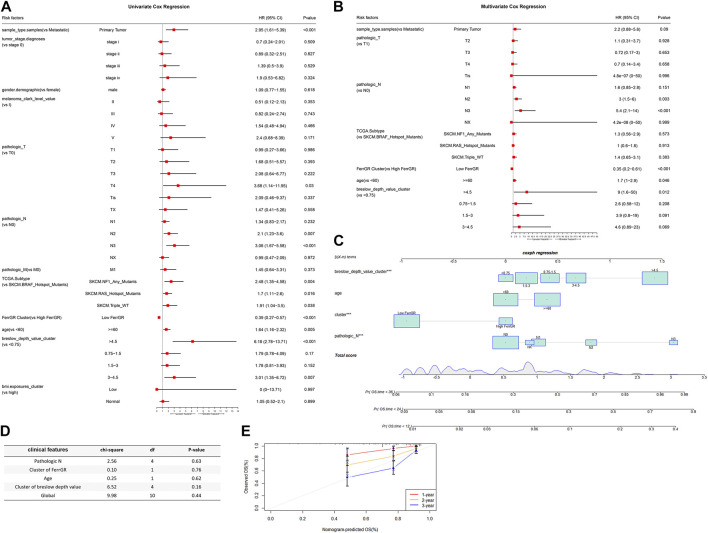
The connection between FerrGR score and conventional clinical characteristics. **(A,B)** Univariate and multivariate regression analysis of FerrGR score and clinical characteristics in prognostic value showed FerrGR score had excellent prognostic independence, **(C)** Prognostic nomogram for SKCM patients with factors, including Breslow depth value, age, FerrGR score and N stage, **(D)** Test for the proportional hazards hypothesis, **(E)** Calibration maps for predicting patient survival at 1, 2, and 3 years. The x-axis and y-axis represent the expected and actual survival rates of the nomogram.

To evaluate whether the risk score was a suitable independent prognostic indicator, univariate and multivariate Cox regression analysis were performed among the clinical characteristics and risk scores in the TCGA cohort. The univariate Univariate Cox regression revealed that clinical parameters, including primary tumor, T4 stage, N2 stage, N3 stage, NF1 mutated subtype, RAS mutated subtype, triple wild type, low FerrGR score, age ≥ 60, Breslow depth value >4.5, Breslow depth value = (3–4.5) were significantly associated with OS (Figure 4A). Through multivariate Cox regression, N2 stage, N3 stage, low FerrGR score, age ≥ 60, and Breslow depth value >4.5 were independent predictors of SKCM (Figure 4B).

What’s more, a nomogram was created based on the values of multiple variables to predict the probability of specific clinical outcomes or events. We constructed the nomogram with the following factors: Breslow depth value, age, FerrGR score, and N stage. In the nomogram, columnar height represents the distribution and number of SKCM patients (Figure 4C). Testing of the proportional hazards hypothesis demonstrated the individual and global variables satisfied the requirement of the hypothesis (Figure 4D). Additionally, the calibration curve for the 1-, 2-, and 3-year survival rates displayed good agreement between the prediction and the investigation (Figure 4E).

### Mutation landscape of FRGs between the high FerrGR group and low FerrGR group

**FIGURE 5:**
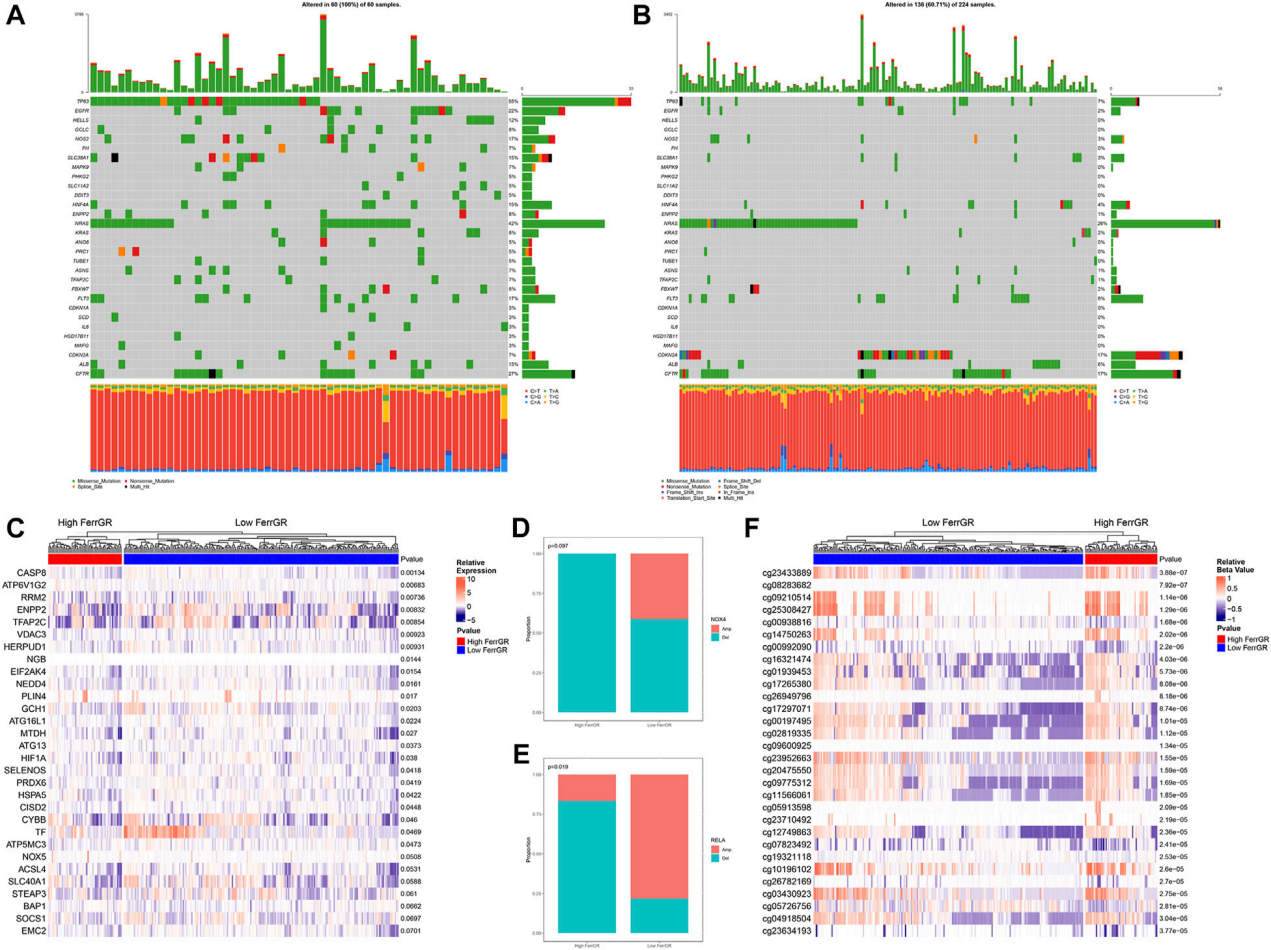
Analysis of ferroptosis-related genomic variation in the high FerrRG group and low FerrRG group **(A,B)** waterfall plots represent mutation information of FRGs in each sample of the high FerrGR group and low FerrGR group SKCM patients, **(C)** Heatmap of top 30 differentially expression FRGs between the high- and the low-FerrGR groups, **(D,E)** The CNV mutation proportion of **(D)** RELA, and **(E)** NOX4 between groups, Amp: Gene amplification, Del: gene deletion **(F)** Heatmap showed the methylation sites of FRGs with top 30 significantly different methylation levels between groups.

Further, the SNV mutation profiles of FRGs in 284 SKCM patients were utilized to explore the different landscape of SNVs in high—and low—FerrRG group patients. Among these patients, 60 belonged to the high FerrRG group and 100% had SNV alterations, while 224 were classified into the low FerrRG group and 136 (60.71%) carried SNV mutations in FRGs. We then collected SNV mutation information in each sample of both groups and presented the top 30 FRGs in Figures 5A,B, respectively. We revealed TP63 (55%), NRAS (42%), CFTR (27%), EGFR (22%), and FLT3 (17%) were the top 5 FRGs with highest mutation frequencies in the high FerrGR group, and NRAS (26%), CFTR (17%), CDKN2A (17%), FLT3 (8%) and TP63 (7%) were top 5 in the low FerrGR group. Notably, missense mutation was the largest fraction of mutation types in both groups.

Next, the differential expression of the top 30 FRGs between the high FerrGR group and the low FerrGR group was exhibited (Figure 5C). We found that CASP8, ATP6V1G1, RRM2, ENPP2, and TFAP2C were the top five differentially expressed genes. CNV analysis then showed RELA and NOX4 were the two FRGs with significantly different CNVs (*p* < 0.1) between high - and Low - FerrGR groups. RELA and NOX4 in the high FerrGR group possessed more widespread CNV deletion (Figures 5D,E). However, there was no significant difference in the CNV status of other FRGs between the two groups (Supplementary Table S3). DNA methylation is an important consideration in the pathogenesis of cancer (McMahon et al., 2017). Therefore, the heat map summarized the 30 most significant FRGs-associated DNA methylation sites between two groups (Figure 5F).

### FerrGR-based prognostic stratification of SKCM patients with immunotherapy

**FIGURE 6:**
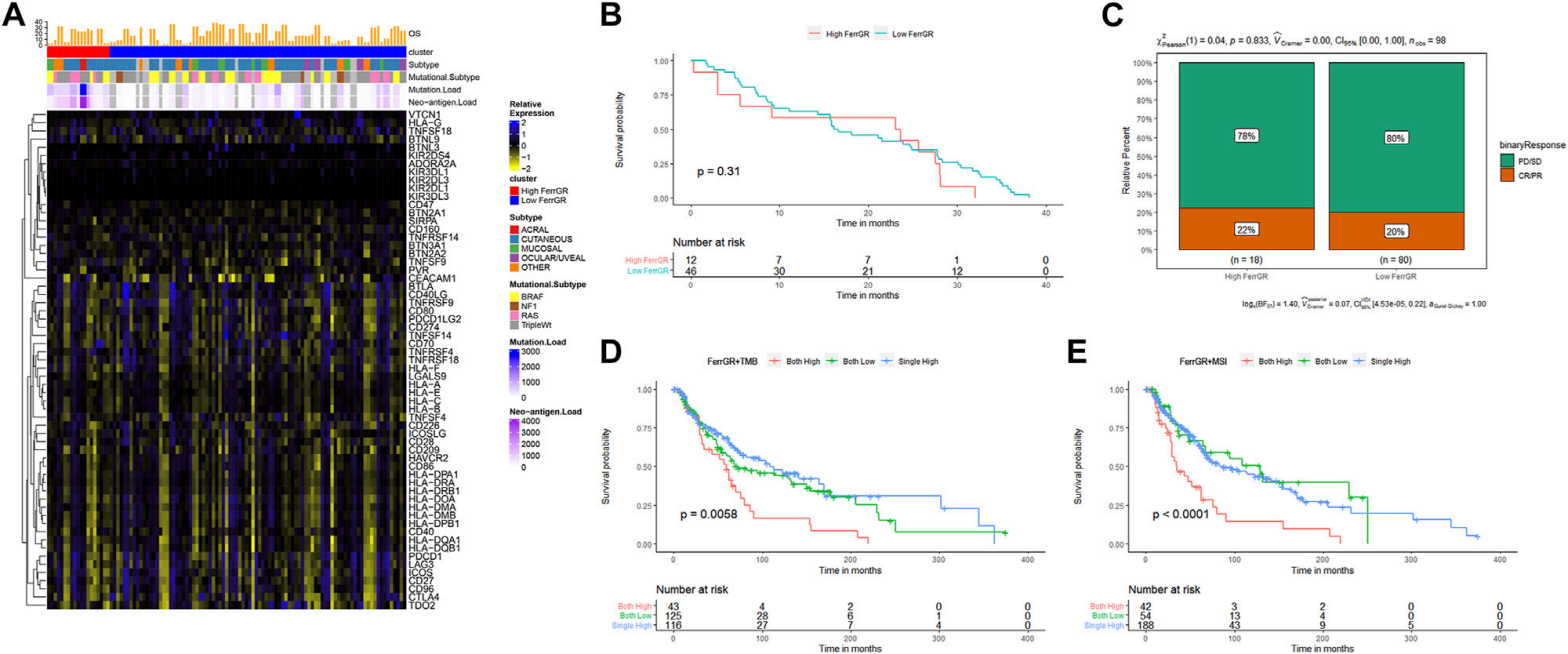
Evaluation of immunotherapy in SKCM with FerrGR model. **(A)** Heatmap displaying the expression levels of 61 immune checkpoint genes in the high FerrGR group and low FerrGR group. **(B)** Kaplan-Meier curves of the high-FerrGR and low-FerrGR group patients in the GSE91061 dataset. **(C)** The proportion of immune response to immunotherapy of high- and low-FerrGR groups in the TCGA cohort. CR, complete response; PR, partial response; SD, stable disease; PD, progressive disease. **(D)** Kaplan - Meier survival analysis of OS among patients within each of the three indicated subgroups (Both high: FerrGR-high/TMB -high; Both low: FerrGR-low/TMB-low; Single high: FerrGR-high/TMB-low or FerrGR-low/TMB-high). **(E)** Kaplan-Meier survival analysis of OS among patients within each of the three indicated subgroups (Both high: FerrGR-high/MSI -high; Both low: FerrGR-low/MSI-low; Single high: FerrGR-high or MSI-high).

Immunotherapy is an innovative treatment strategy for cancers. In particular, immune checkpoint blockade (ICB) therapy has made great progress in immunotherapy for cancer patients (Havel et al., 2019). Hence, we firstly determined the differences in the expression levels of 61 immune checkpoints between the high FerrGR group and low FerrGR group of the GSE91061 dataset (Figure 6A). We then revealed that there was no significant difference in patient OS between these two groups (Figure 6B). Subsequently, the response to immunotherapy was studied and found that no significant difference in immunotherapy responses between the high FerrGR group (*n* = 18) the and low FerrGR group (*n* = 80), implying that the FerrGR model may not be a direct biomarker of immunotherapy (Figure 6C). Thus, we further investigated the joint utility of FerrGR combined with TMB or MSI for patient stratification and prediction of clinical outcomes. The FerrGR-high/TMB-high and FerrGR-high/MSI-high (both high) subgroups had a remarkably poorer survival outcome compared with the subgroups where both were low or single was high (Figures 6D,E). These results demonstrated that a combination of FerrGR and TMB/MSI served as a combined biomarker with better predictive value for favorable ICIs benefit.

### Identification of the relationship between the FerrGR model and tumor immune microenvironment

**FIGURE 7:**
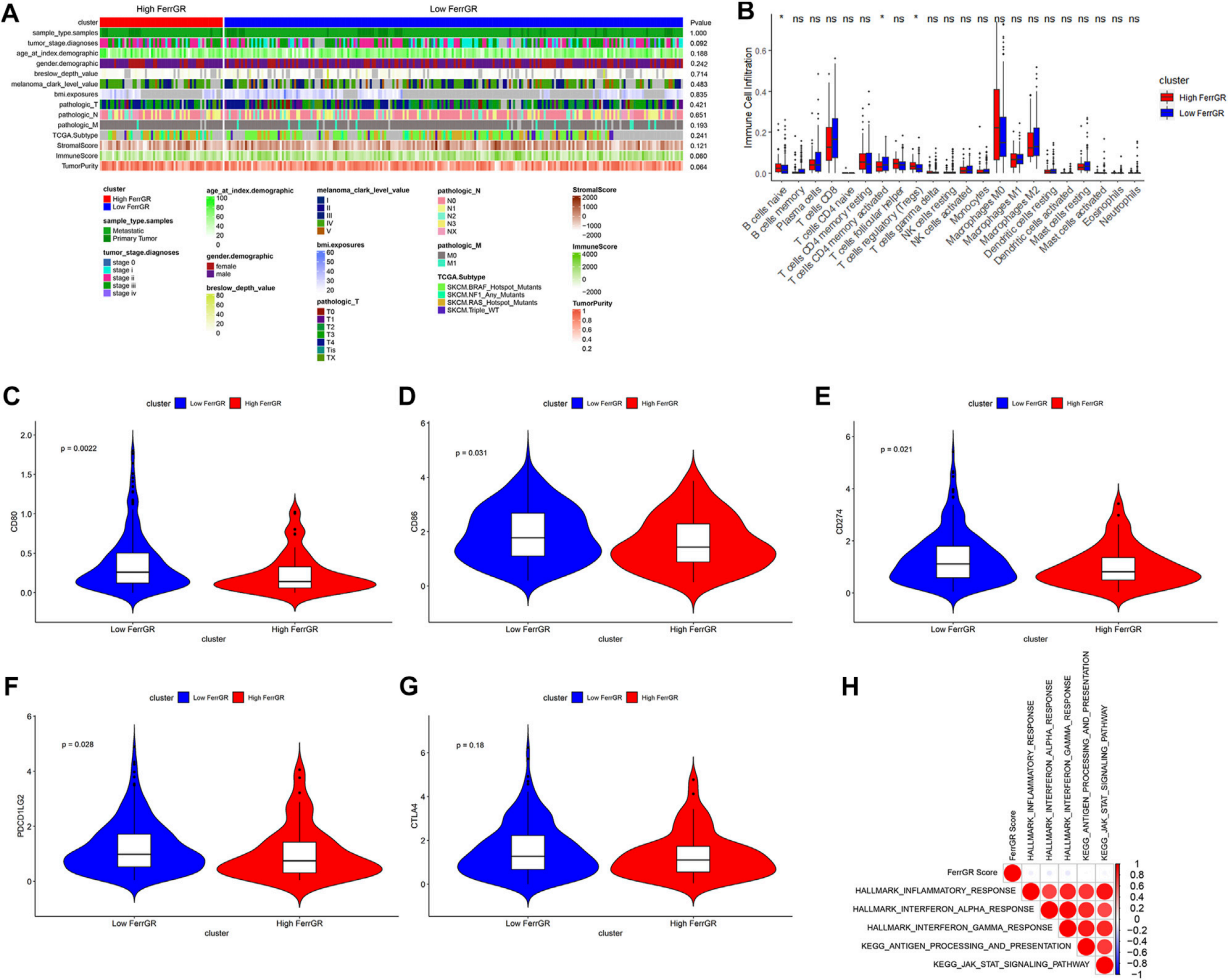
Tumour immune microenvironment analysis of FerrGR model. **(A)** The heatmap showed the correlations between FerrGR score and clinicopathological features. **(B)** Boxplot exhibited the distribution patterns of immune cell infiltration between two FerrGR groups. **(C–G)** violin plots visualizing the expression of immune checkpoint genes in the high—and Low—FerrGR groups, **(C)** CD80, **(D)** CD86, **(E)** CD274, **(F)** PDCD1LG2, **(G)** CTLA4 **(H)** Gene set enrichment analysis (GSEA) results depicting enrichment of immune-related pathways based on FerrGR score.

To better study how the FerrGR model and the immune microenvironment interact, we firstly evaluated the different distribution of clinicopathological features between two FerrGR group patients of the TCGA cohort, and revealed that patients in the high FerrGR group had a lower immune score, higher tumor grade, and higher tumor purity than in low FerrGR group (Figure 7A). The distribution patterns of 22 immune cells between two groups were next calculated by the CIBERSORT algorithm. The comprehensive comparisons with the FerrGR score showed that B cells naive and T cells regulatory (Tregs) were enriched in the high FerrGR group obviously, while the patients in the low FerrGR group had a higher level of T cells CD4 memory activated (Figure 7B). It is known that immune checkpoint genes usually make an immunosuppressive effect in tumorigenesis and immune evasion. Therefore, the expression levels of immune checkpoint genes in high—and low—FerrGR groups were compared, and the results indicated that the expression levels of common immune checkpoint genes, including CD274 (PD-L1), CD80, CD86 and PDCD1LG2 (PD-L2), in the low FerrGR group were all higher than those in the high FerrGR group (Figures 7C–F). However, there was no remarkable differential expression of CTLA4 between these two groups (Figure 7G). Subsequently, GSEA was performed to determine the biological functions and signal transduction pathway associated with the FerrGR score. The results showed that the FerrGR score was negatively correlated with inflammatory response, interferon-alpha response, interferon-gamma response, antigen processing and presentation, and the JAK-STAT signaling pathway, respectively (Figure 7H). These findings revealed that SKCM patients with high FerrGR scores prefer to form a suppressive immune microenvironment by increasing suppressive immune infiltration cells and upregulating immune checkpoint genes.

### Potential sensitive drugs for SKCM according to the FerrGR model

**FIGURE 8:**
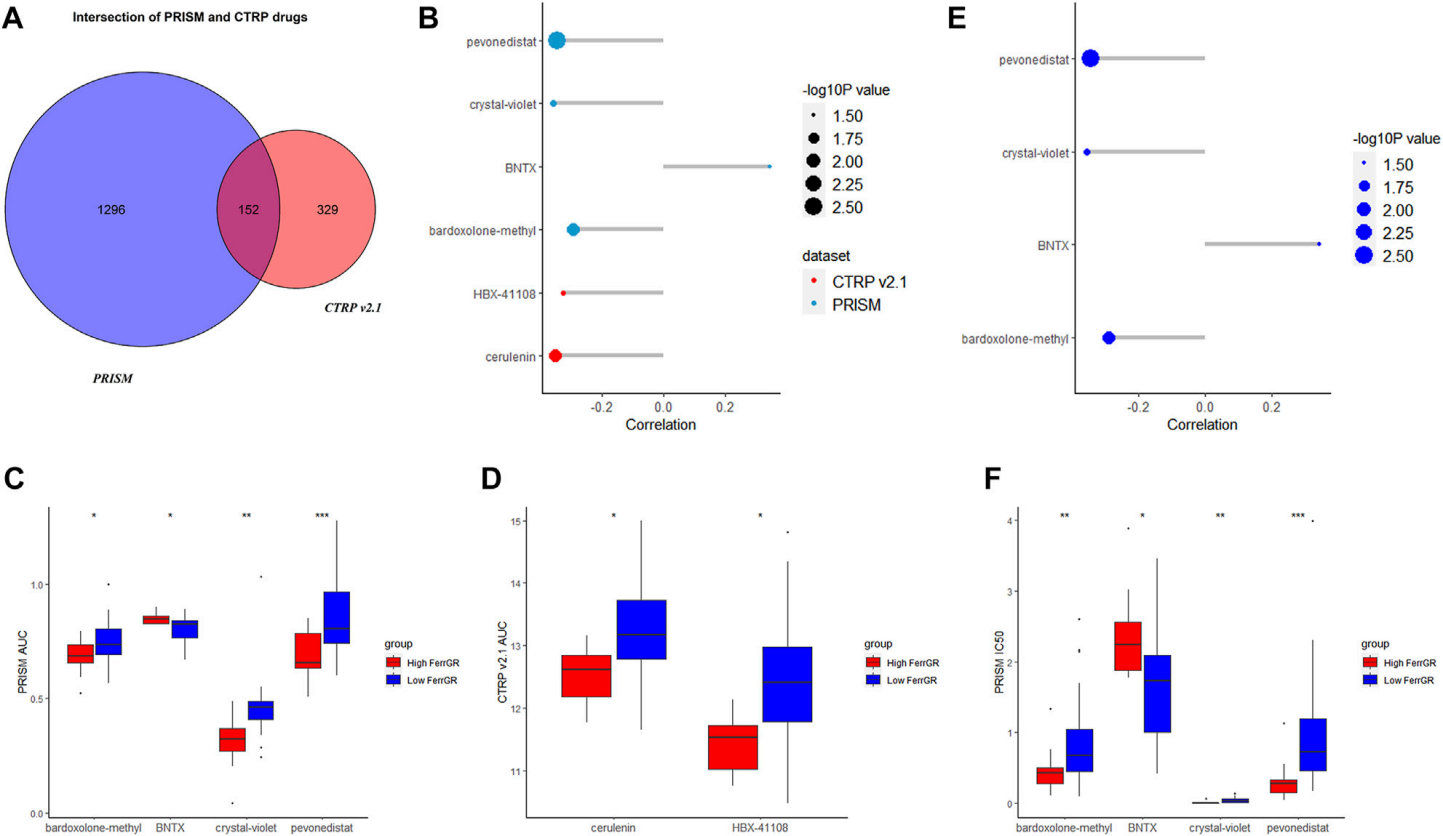
Potential targeted drugs prediction on basis of the FerrGR model. **(A)** The Venn chart showed the number of drugs in the PRISM dataset and CTRP v 2.1 databases. **(B)** The correlation between the AUC value of potential drugs and the FerrGR score. **(C,D)** The distribution of the AUC value of each potential drug from **(C)** RISM dataset and **(D)** CTRP v 2.1 database according to the FerrGR model. **(E)** The correlation between the IC50 value of potential drugs and the FerrGR score. **(F)** The distribution of the IC50 value of each potential drug on basis of the FerrGR model.

According to the data on drug sensitivity and expression, 1,311 and 481 potential sensitive compounds were figured out from the PRISM and CTRP database respectively, and 152 overlapped compounds were filtered out (Figure 8A). It was accepted that values of AUC and IC50 represented the sensitivity of the cells to drugs and were negatively correlated with the sensitivity. We then identified the top differential AUC value and IC50 value between high—and low—FerrGR group samples, and determined a threshold to select potential compounds. The Spearman’s correlation >0.2 was set as the threshold. The AUC values of pevonedistat, crystal-violet, bardoxolone-methyl, BNTX from the PRISM database, and cerulenin, HBX-41108 from the CTRP database exhibited significant correlations with the FerrGR score. Apart from BNTX showing a positive correlation with the FerrGR score, the other five selected compounds had negative correlations (Figure 8B). Besides, differential distribution of the AUC value of six potential compounds in high—and low—FerrGR groups was depicted (Figure 8C, D). Similarly, pevonedistat, crystal-violet, bardoxolone-methyl, and BNTX were identified from the PRISM database based on their IC50 values, and no potential compounds were found in the CTRP database. BNTX had a positive correlation with the FerrGR score, while pevonedistat, crystal-violet, and bardoxolone-methyl had negative correlations (Figure 8E). The differential distribution of the IC50 value of these four potential compounds in high—and low—FerrGR groups was exhibited in Figure 8F. Therefore, those compounds may be novel options for SKCM treatments in the future.

## Discussion

SKCM is highly heterogeneous in the genetic, epigenetic, and gene expression with high metastases and death threats (Grzywa et al., 2017; Hendrix et al., 2017). Understanding the rapid progression of this heterogeneity makes possible the molecular classification and individualized treatment of SKCM. Ferroptosis has gained the interest of numerous researchers due to its unique cell death mechanism and its potential therapeutic prospects in cancers (Jiang et al., 2021). Current studies have constructed several prognostic prediction models for SKCM based on the expression of FRGs. Zeng et al. developed a prognostic model depending on the expression of two FRGs (ALOX5, CHAC1), and differences in the underlying diseases of SKCM did not effect on the expression features of these two genes (Zeng et al., 2021). Additionally, studies showed five-, six-, eight-, nine- and ten- FRG predictive models according to the RNA sequencing data have been constructed (Xu Z et al., 2021; Xu and Chen, 2021; Chen et al., 2022; Ping et al., 2022; Yue et al., 2022). These models forecasted the melanoma patients’ prognosis and exhibited the close relationship between immune function and FRGs. However, the role of ferroptosis in SKCM patients, especially the mechanism of the association and interaction between ferroptosis-related genetic mutations and the clinical outcomes is still unclear. After performing a series of bioinformatics analyses, we found that SNVs of FRGs are an indicator of prognosis and TME status in SKCM patients, which may be of great significance for future research. Therefore, based on the SNV landscape, we systematically identified FRGs with prognostic ability to establish a robust and accurate ferroptosis-associated genetic mutation risk model to predict prognosis in SKCM patients and illustrate the relationship between ferroptosis-related SNVs and the TME.

In this study, the TCGA-SKCM cohort was used to perform univariate Cox regression combined with the previously reported and identified 24 FRGs that were correlated with SKCM prognosis. Subsequently, the LASSO algorithm was used to reduce dimensionality and construct a 19-gene signature prognostic model (FerrGR model). We verified the effectiveness of this model in the training cohort and the validation cohort. The FerrGR score of each sample is calculated on basis of whether the sample has SNV mutations in the 19 key genes or not. Then, patients in the training cohort and validation cohort were classified into the high FerrGR group and the low FerrGR group. The results showed that it is an independent, effective and robust prognostic model in both cohorts where the prognosis was worse in the high FerrGR group. In addition, aiming at the characteristics of high heterogeneity in SKCM patients, we established and validated a nomogram based on FerrGR score and clinicopathological indications that can predict 1-, 2-, and 3-year OS for individual SKCM patients specifically.

Nowadays, high-throughput sequencing technologies allow us to detect numerous genes which are significantly related to melanoma prognosis through comprehensive analyses and establish multiple biomarkers. Therefore, recent studies explored novel favorable prognostic genes, such as aging-related genes, metabolic genes and pyroptosis-related genes, to predict prognosis and immune response for SKCM (Ju et al., 2021; Guo et al., 2022; Zeng et al., 2022). However, most of these studies did not systematic and in-depth analysis of the genetic mutations of these prognostic genes. SNVs are somatic point mutations found in cancer tissues and enriched in cancer driver genes and cellular pathways which are essential for tumorigenesis. Tumorigenesis is an evolutionary process of accumulation of somatic mutations (driver mutations), which promotes a selective growth advantage for cancer cells (He et al., 2014). Several pathogenic CNVs in special genes have been reported in the beginning and development of breast cancer subtypes, including BRCA1, MTUS1, and hTERT, suggesting that CNVs also play a unique role in breast cancer (Frank et al., 2007; Silva et al., 2014). Here, we found that the proportion of SNV mutations in ferroptosis-related genes associated with SKCM was 82.17% (235/286), among which the SNV mutation frequency of NRAS (20%), CFTR (19%), and TP63 (18%) ranked the top three. SNV mutations in FGRs might play an important role in the development and progression of SKCM. Meanwhile, the SNV mutation frequency of FGRs in the high FerrGR group was higher than in the low FerrGR group, especially TP63 and NRAS. TP63 mutations, which are present in the majority of cancers, are associated with poorer clinical outcomes in SKCM (Matin et al., 2013; Monti et al., 2017), which is consistent with our findings. The relationship between TP53 and NRAS mutational status and SKCM survival was substantially more pronounced. NRAS mutations are discovered in 15% of SKCM cases and more likely to have an aggressive tumor (Kelleher and McArthur, 2012; Muñoz-Couselo et al., 2017). In addition to SNV mutations, CNV mutations were also assessed in this study. However, the notable differential CNV variants between high—and low—FerrGR groups were only discovered in RELA and NOX4. It is consistent with literature reports that SKCM mainly had SNV mutations but rarely CNVs (Liu et al., 2020). We also discovered that two FerrGR groups have their unique methylation levels. Overall, these results indicated that there were differences in expression and variation of FRGs between the two FerrGR groups. Besides, SNVs are likely to be the reason why the FRGs are ferroptosis resistance genes.

ICIs, such as anti-PD-1 and anti-PD-L1 antibodies, exert their effects by releasing the braking effect of the anti-tumor response immune system. Despite the breakthrough in ICIs therapy, it did not work as well in all patients. Therefore, there is a strong interest in finding biomarkers that can identify good responses to ICIs treatment. Several studies showed the risk of TMB and neoepitopes had a close correlation with immunotherapy. High TMB contributed to low survival outcomes and lower SKCM immune infiltrates (Jiang F et al., 2020). In addition, tumors having a high mutation load tend to respond to PD-1 immunotherapy more quickly and have a better prognosis (Samstein et al., 2019). There are still some patients with high TMB who do not respond and *vice versa*. The important reasons are that TMB only focuses on the number of mutations, and the current TMB calculation method gives the same weight to each gene mutation, which is not precise enough to define the overall pattern of anti-tumor immune response (Sha et al., 2020). MSI is often caused by a mismatch repair deficiency (MMR), and patients with MMR have extremely high rates of TMB (Nakayama et al., 1983). These tumors also have a significant response to immunotherapy (Le et al., 2015; Le et al., 2017). However, MMR is uncommon in melanoma (Tomlinson et al., 1996; Richetta et al., 1997; Birindelli et al., 2000). Unfortunately, the model in this study cannot be used as an independent prognostic factor for immunotherapy. Therefore, we explored the effect of the FerrGR model in combination with TMB or MSI for patient stratification and prediction of clinical outcomes, and found that patients with high-FerrGR/high-TMB, and patients with high-FerrGR/high-MSI, had the worst outcomes. Although other literature suggests that SKCM patients with high TMB or high MSI have a better prognosis (Sha et al., 2020), our results showed that these patients had a worse prognosis when the FerrGR score was high at the same time. Therefore, we hypothesized that the FerrGR score was a co-predictor with TMB and MSI, and that the FerrGR model could enhance the predictive ability of TMB and MSI.

The TME is made up of tumor cells and non-tumor cells that play a vital role in tumor growth and progression (Lim and South, 2014). Immune cells and stromal cells are two major types of non-tumor components in the TME. According to recent research, tumor progression can be caused by imbalances between tumor progression and the host immune response (Galon et al., 2013). Of note, ferroptosis can promote tumor growth by driving the polarization of macrophages in the TME (Dai et al., 2020). Moreover, hypoxia-inducible factor (HIF) pathways are a positive trigger for ferroptosis in clear-cell carcinoma (CCC) (Zou et al., 2019). Based on the ESTIMATE algorithm, we analyzed the relationship between the two groups and clinical features and assessed immune cell infiltration in TME. Compared with the low FerrGR group, the high FerrGR group had a higher immune score and higher tumor purity. Generally, immune scores increased significantly with the malignant progression of SKCM (Ning et al., 2021), while tumor purity decreased at higher grades in our analysis. Immune cells constitute a comfortable environment for tumor growth, suggesting that the poor prognosis of patients in the high FerrGR group is due to the tumor immunosuppressive environment (TIME). TIME is the immunosuppressive part of TME, which consists of immunosuppressive cells and immunosuppressive cytokines. Ferroptosis-related genes with a higher frequency of SNV mutations in the high FerrGR group may be associated with increased infiltration of immunosuppressive cells in SKCM. Tregs regulate innate and adaptive immune cells and maintain self-tolerance (Sakaguchi et al., 2008). A high proportion of Tregs is associated with tumor progression, poor survival in many solid tumors, including SKCM (Gerber et al., 2014), and poor clinical outcomes in SKCM patients treated with immunotherapy (Cesana et al., 2006). Here, our studies supported that a high proportion of Tregs in the high FerrGR group existed antitumor immune responses mediated by T cells. Conversely, the proportion of T cells CD4 activated in the low FerrGR group contributed more to immune response than in the high FerrGR group, according to our findings. Interestingly, we also found that almost all immune checkpoint genes, including PD-L1 and PD-L2, were upregulated in the high FerrGR group. Collectively, these results may be a sign of immune escape in the high FerrGR group patients.

Six potential targeted drugs, including pevonedistat, crystal-violet, bardoxolone-methyl, BNTX, cerulenin and HBX-41108, for high FerrGR samples were predicted. Pevonedistat (MLN4924) leads to DNA re-replication, cell cycle arrest and death *via* targeting the NEDD8-activating enzyme (NAE). It has anti-tumor activity and supports the clinical benefits observed in recent clinical trials in SKCM patients (Wong et al., 2017; Wood et al., 2020). For immunotherapy, the combination of pevonedistat and anti-PD-L1therapy had a better therapeutic efficacy compared to each agent alone. Pevonedistat attenuated T cell killing through PD-L1 induction, whereas blockade of PD-L1 successfully potentiated the sensitivity of pevonedistat-treated glioblastoma cancer cells to T cell killing (Zhou et al., 2019). Hence, a combination of pevonedistat with immune checkpoint blockade treatment might be promising combinatorial regimens. Bardoxolone methyl is a novel synthetic triterpenoid and antioxidant inflammation modulator that activates Nrf2 and inhibits NF-κB. It can impair tumor growth and induces radiosensitization of oral squamous cell carcinoma cells (Hermann et al., 2021). But a further examination of its effects in SKCM is required. Cerulenin, a fatty acid synthase inhibitor, can retard the growth of melanoma cells and activates caspase-dependent apoptosis (Ho et al., 2007). Moreover, the anti-tumor immune responses of cytotoxic T cells were potentiated and ovarian tumor growth was inhibited by treatment with cerulenin (Yoon and Lee, 2022). It indicated that cerulenin might have potential applications in cancer immunotherapy. HBX-41108 is a partially-selective ubiquitin-specific proteases (USPs) inhibitor that stabilizes p53 and induces caspase 3 and PARP cleavage in cancer cells. As USPs are therapeutic targets for tumor treatment, HBX-41108 is likely to be an effective drug for SKCM (Pal and Donato, 2014). Therefore, the relationship between these potential targeted drugs and SNV mutations, ferroptosis, SKCM progression and immunotherapy needs further exploration.

Taken together, our results suggested that the FerrGR model based on SNV mutations of 19 key FRGs is a reliable prognostic risk prediction model for predicting the overall survival of SKCM patients. This may help guide treatment strategies for SKCM to improve clinical outcomes and provide theoretical references for explaining the prognosis difference between patients. Nevertheless, our study has several limitations. Firstly, there are not relatively abundant key FRGs in the risk model, which may limit it for clinical application. In addition, there was no significant difference between the two FerrGR groups in the immunotherapeutic response, thus more prospective real-world data should be used to confirm the accuracy and applicability of this model. Besides, further validation of this model in prospective studies of SKCM patients is needed.

## Conclusion

In a word, we developed the FerrGR model for predicting the clinical outcomes and guiding the treatment of SKCM. It might have a contribution to distinguish immune and molecular features, stratify SKCM patients benefiting from immunotherapy, predict patient survival, and discover potential targeted drugs Our study provides new insights into genetic mutations of FRGs in SKCM’s development and progression, and offers novel ideas for advancing the treatment of SKCM by targeting ferroptosis. However, further research on confirming the prognostic value of the FerrGR model is required.

## Data Availability

The original contributions presented in the study are included in the article/Supplementary Material, further inquiries can be directed to the corresponding authors.

## Abbreviations

AUC, areas under the curve; CNVs, copy number variations; FerrGR, ferroptosis-related genetic mutation risk; GSVA, gene set variation analysis; ICIs, immune checkpoint inhibitors; LASSO, least absolute shrinkage and selection operator; MMR, mismatch repair deficiency; MSI, microsatellite instability; OS, overall survival; PD-L1, programmed cell-death receptor 1 ligand; ROC, receiver operating characteristic; SNV, single-nucleotide variant; TMB, tumor mutational burden; TIME, tumor immunosuppressive environment; TME, tumor microenvironment.
