# Supplementary figures and images for: A new ferroptosis-related genetic mutation risk model predicts the prognosis of skin cutaneous melanoma
Source: Front Genet. 2023 Jan 5;13:988909. doi: 10.3389/fgene.2022.988909 (PMC9849373; doi:10.3389/fgene.2022.988909)

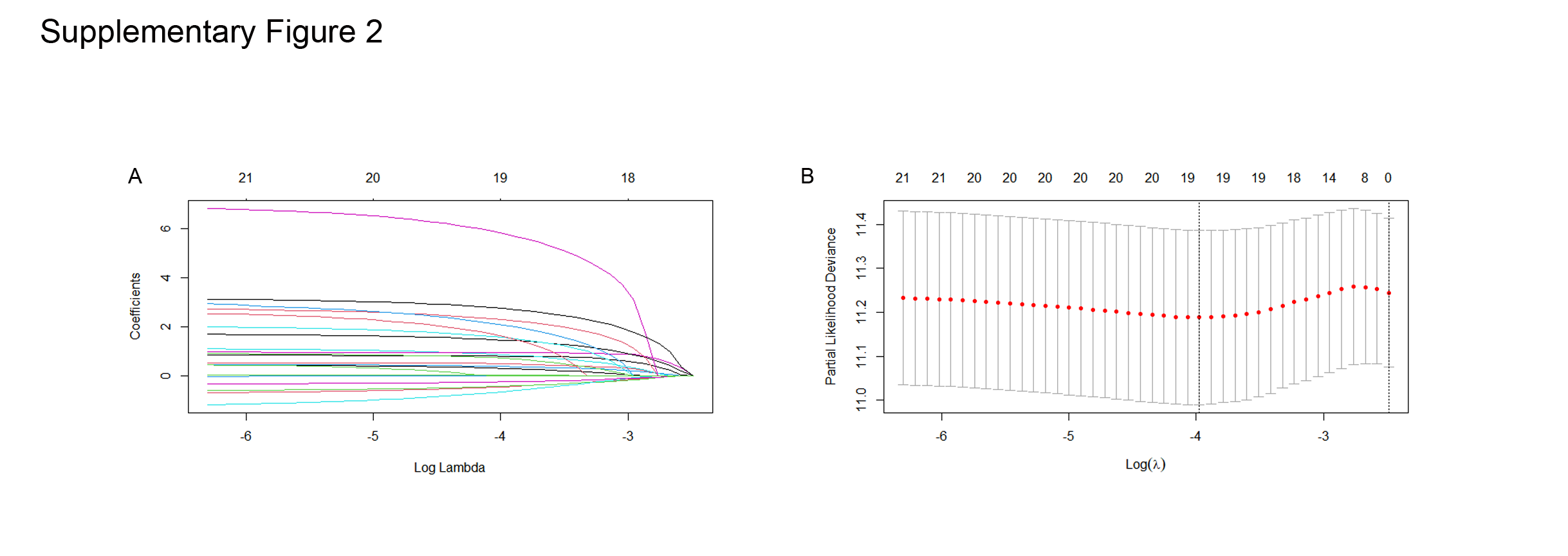

Supplement: Supplementary file 3 [file Image2.TIF]

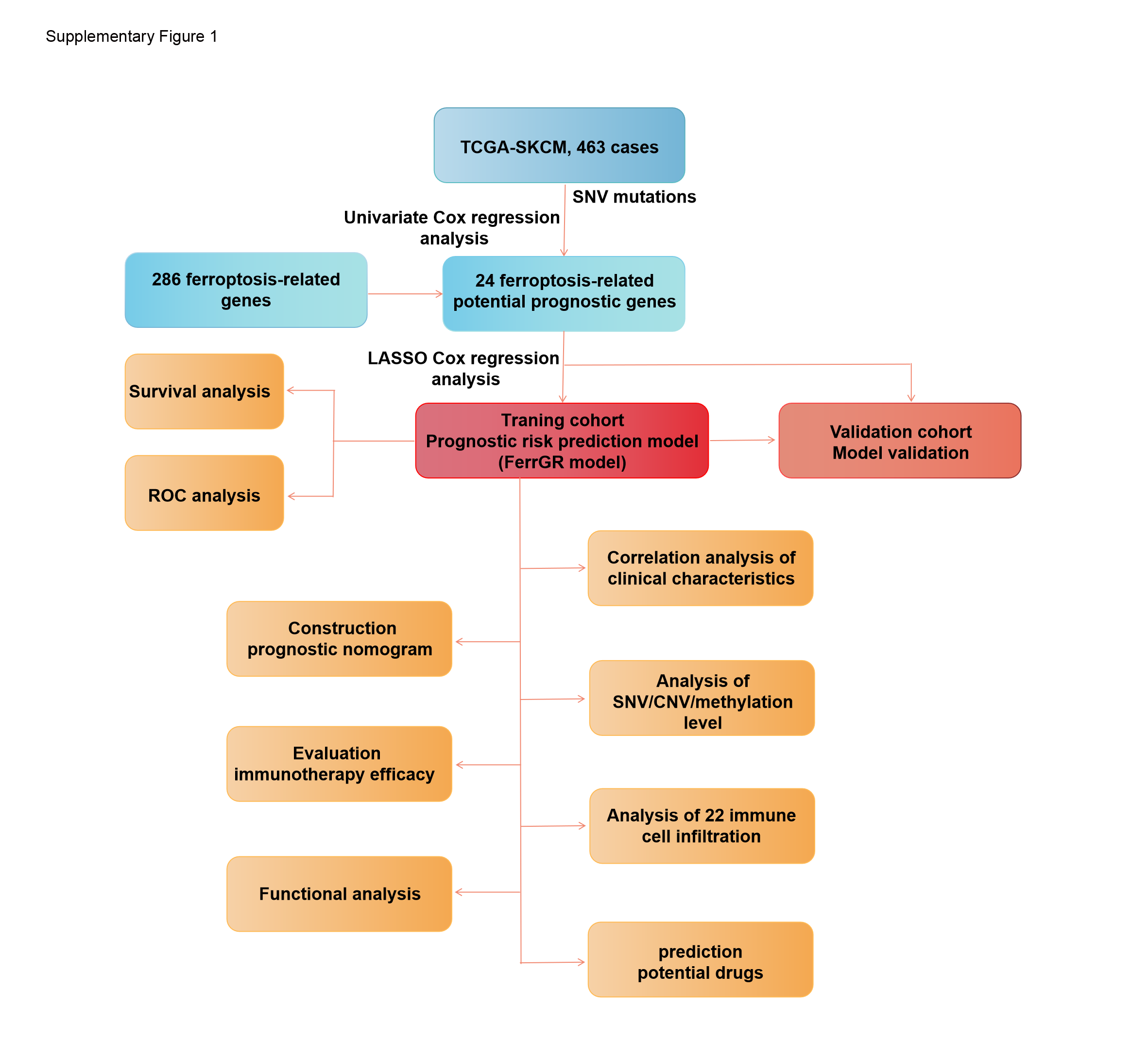

Supplement: Supplementary file 4 [file Image1.TIF]
